# How do medical students differ in their interpersonal needs?

**DOI:** 10.1186/s12909-017-0870-y

**Published:** 2017-02-21

**Authors:** Yera Hur, A Ra Cho, Sun Huh, Sun Kim

**Affiliations:** 1 Department of Medical Education, Konyang University College of Medicine, Daejeon, Republic of Korea; 2 Department of Medical Education, College of Medicine, The Catholic University of Korea, Seoul, Republic of Korea; 3 Department of Parasitology and Institute of Medical Education, College of Medicine, Hallym University, Chuncheon, Republic of Korea

**Keywords:** Personal characteristics/attitudes, Physician/patient relationship, Interpersonal education

## Abstract

**Background:**

Knowing one’s interpersonal relationship preferences can be tremendously helpful for medical students’ lives. The purpose of this study was to examine the interpersonal needs in medical students.

**Methods:**

Between 2010 and 2015, a total of 877 students from four Korean medical schools took the Korean version of the Fundamental Interpersonal Relations Orientation – Behaviour (FIRO-B) scale. The FIRO-B results were analyzed by descriptive statistics, frequency, independent *t*-test, and one-way ANOVA.

**Results:**

The medical students’ scores for interpersonal needs were moderate overall, with the highest scores for control (M = 8.63, SD = 3.08), followed by affection (M = 8.14, SD = 4.34), and inclusion (M = 7.81, SD = 4.30). Gender differences showed in three areas: expressed control (male > female, t = 4.137, *p* < 0.001), wanted affection (male < female, t = −3.148, *p* = 0.002), and control needs (male > female, t = 2.761, *p* = 0.006). By school type, differences were shown in expressed control (t = 3.581, *p* < 0.001), wanted inclusion (t = 2.625, *p* = 0.009), Inclusion (t = 1.966, *p* = 0.050), and expressed (t = 2.077, *p* = 0.038); undergraduate medical college (MC) students’ needs were greater than the needs of graduate medical school (MS) students, but for wanted control, the MS students showed greater needs (t = −2.122, *p* = 0.034).

**Conclusions:**

There were differences in all categories except for expressed inclusion, wanted control, and control. The FIRO-B is a useful tool for giving insight into students regarding their interpersonal orientations, which will help them to adjust to medical school life. In addition, the FIRO-B can be useful when mentoring and coaching students.

## Background

We all know that a good patient-doctor relationship is essential to modern health care. There are many approaches to building good patient-doctor relationships, and many medical school curricula provide communication skills courses to excel such competencies [1–3]. We approached the subject in a different way. We evaluated medical students’ interpersonal relationship orientations using the Fundamental Interpersonal Relations Orientation-Behavior (FIRO-B) inventory by Schutz (1958, as cited in Young [4]).

The first step in building good patient-doctor relationships is to help medical students to be aware of who they are and what kinds of interpersonal orientations they have. It is important for medical students to know and understand the differences in human behavior in interpersonal relationships both because they will work with diverse patients in the future and this knowledge will also help them to work well with their colleagues. Moreover, as Reis et al. [5] observed, knowing one’s interpersonal relationship type is essential because it is the foundation of human life.

The tool we used in this study, the FIRO-B, is widely used for interpersonal relationships [4], but not in medical education. Using the keyword “FIRO-B” in all fields in PubMed Central revealed only two results related to Shutz’s assessment tool for interpersonal relationships [6], and these papers did not address medical education or medical students. Two previous studies that used the instrument in medical education can be found in the *Journal of Korean Medical Education* [7, 8], but these studies focused on comparing the FIRO-B with other personality inventories or finding correlations between the FIRO-B and stress or depression. These few attempts had some significance, but very little.

This study has its originality in that it intended to focus solely on the interpersonal orientations of medical students and to properly introduce the special features of a simple instrument, the FIRO-B. The following are the three overarching topics this study examined: 1) What are the medical students' fundamental interpersonal relationship behaviors? 2) Are there differences in interpersonal needs between genders? 3) Are there differences in interpersonal needs by school system? and 4) Are there differences in interpersonal needs by academic level?

## Methods

### Participants

Four medical schools in Korea participated from 2010 to 2015. One of the participating schools had both an undergraduate medical college and a graduate medical school. In Korea, undergraduate medical college (MC) takes six years and graduate medical school (MS) takes four years. Nine hundred twenty-one medical students took the FIRO-B, 44 surveys were excluded for missing data, so a total of 877 results were used (Premedical year 1: male = 231, female = 140, Premedical year 2: male = 52, female = 19, Medical year 1: male = 188, female = 142, Medical year 2: male = 65, female = 40). Five hundred sixty-nine students were from of the MC and 308 were from the MS (Table 1).

**Table 1:** Number of students by school type and academic level

			School type					Total
			Undergraduate medical college			Graduate medical school		
			A-1	B	C	A-2	D	
Academic Level	Premedical Year 1	Male	40	191	-	-	-	231
		Female	24	116	-	-	-	140
	Premedical Year 2	Male	-	-	52	-	-	52
		Female	-	-	19	-	-	19
	Medical Year 1	Male	-	-	52	81	55	188
		Female	-	-	16	87	39	142
	Medical Year 2	Male	-	-	46	-	19	65
		Female	-	-	13	-	27	40
Total			569			308		877

Data are presented as number

### Material

The FIRO-B was first introduced by Schutz in 1958 [4], and the Korean version consists of 54 items. The reliability of the instrument using coefficient reproducibility ranged from 0.88 to 0.96 for the six individual needs categories: expressed-inclusion (eI) = 0.90, wanted-inclusion (wI) = 0.96, expressed-control (eC) = 0.93, wanted-control (wC), wanted-affection (wA) = 0.95, and expressed-affection (eA) = 0.88. Interpersonal needs consists of three areas, inclusion, control, and affection, and behavior is measured in two aspects, expressed and wanted. These measurements are combined to form the six individual needs above: eI, eC, eA, wI, wC, and wA. The items are ranked from 1 (never) to 6 points (always), so that the overall needs score ranges from 0 to 54 points maximum. The three needs dimensions, inclusion, control, affection, are scored between 0 and 18, and the scores of the two behavior aspects, expressed and wanted, are scored between 0 and 9 [4]. Table 2 shows the specific score ranges and briefly describes each need and behavior.

**Table 2:** FIRO-B score range

Score range	Low	Medium	High
Needs	0–5	6–12	13–18
	♦ Inclusion: Being part of a group, recognition ♦ Control: Influencing situations, leading, responsibility ♦ Affection: Being close with individuals, building rapport		
Behaviors	0–7	8–19	20–27
	♦ Expressed: What you tend to do; how much you initiate this behavior with others; observable action ♦ Wanted: How much you tend to want others to initiate this behavior with you; how much you prefer to be the recipient		
Six dimensions of needs	0–2	3–6	7–9
Overall	0–15	(Low) 16–26 (High) 27–38	39–54

### Statistical analysis

The medical students’ interpersonal needs score and distribution were analysed with descriptive statistics for mean scores and frequency. Difference by gender and school system were analysed with independent *t*-test. One-way ANOVA of parametric statistical tests was used to identify the significant differences between academic years. All tests were two tailed, and *p*-value of <0.05 was considered significant. Statistical analysis was done using SPSS version 21.0 (IBM Corp., Armonk, USA).

## Results

### Level of interpersonal needs

**Fig. 1:**
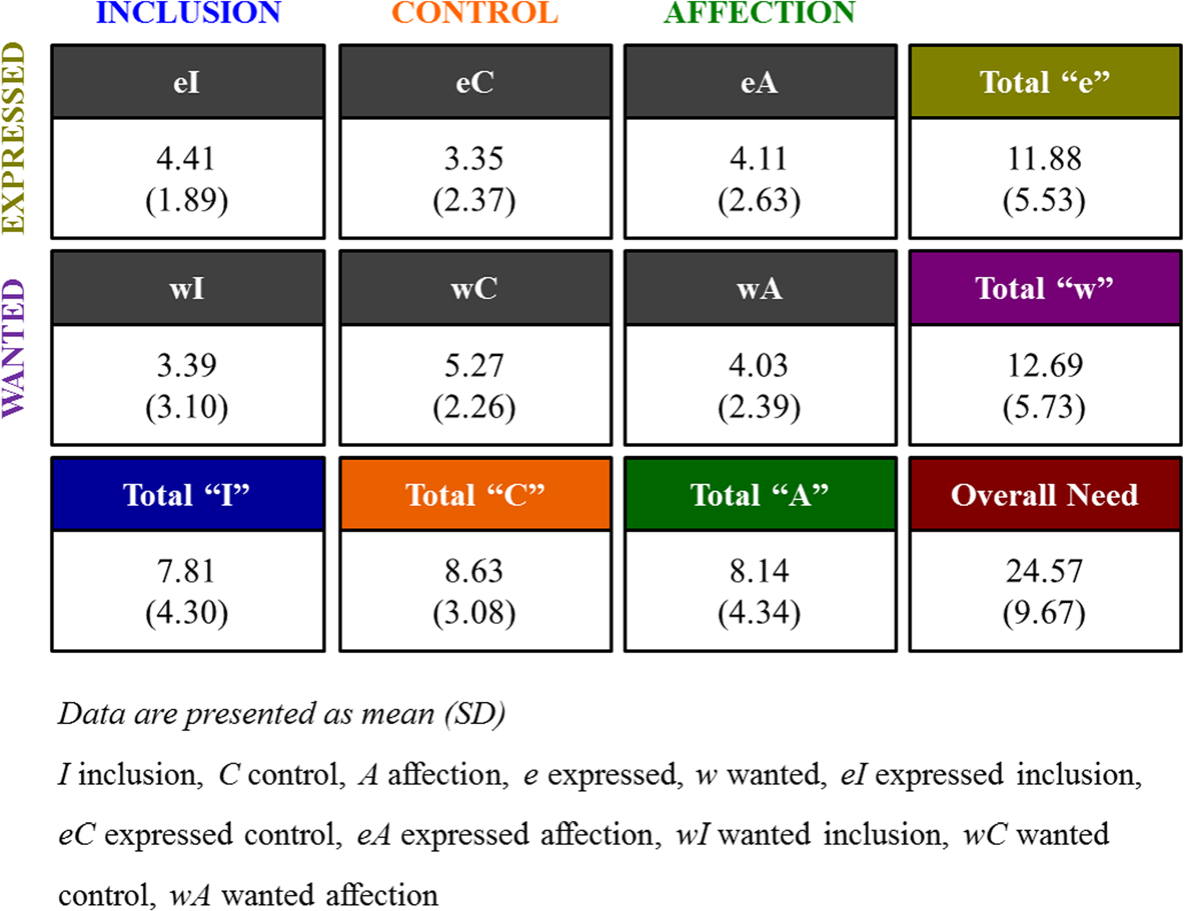
FIRO-B Scores

**Fig. 2:**
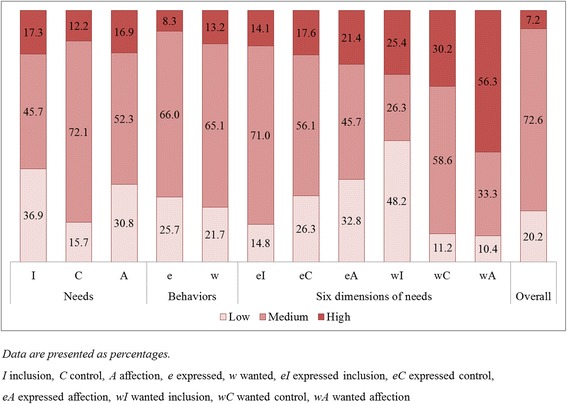
Distribution Scores for Interpersonal Relationship Needs

The medical students' FIRO-B results are shown in Figs. 1 and 2. The medical students’ interpersonal needs scores as moderate overall; specifically, control’ needs were the highest (8.63 ± 3.08) followed by affection (8.14 ± 4.34) and inclusion (7.81 ± 4.30). Expressed and wanted behavior levels were also moderate (e 11.88 ± 5.53, w 12.69 ± 5.73). By needs level, many of the medical students had high wA scores (56.3%), and 48.2% of students showed low wI scores.

### Differences by gender

Gender differences were analyzed, and the results showed statistically significant differences in three areas. From the six dimensions of needs, males showed higher expressed control needs than did females (Male 3.61 ± 2.45 > Female 2.95 ± 2.19; t = 4.137, *p* < 0.001), and female showed higher wanted affection than did male (Female 4.34 ± 2.47 > Male 3.82 ± 2.32; t = −3.148, *p* = 0.002). The only difference in the needs category was found for control; specifically, males had greater control needs than did females (Male 8.85 ± 3.23 > Female 8.28 ± 2.78; t = 2.761, *p* = 0.006).

### Differences by school type

Korea has a four-year MS and a six-year MC system that includes two years of premedical courses. Comparing these two school systems, in which MS students are much older than MC students, differences were found in eC (MC 3.56 ± 2.31 > MS 2.97 ± 2.45; t = 3.581, *p* < 0.001), wI (MC 3.59 ± 3.15 > MS 3.03 ± 2.97; t = 2.625, *p* = 0.009), I (MC 8.01 ± 4.41 > MS 7.43 ± 4.08; t = 1.966, *p* = 0.050), and e (MC 12.16 ± 5.48 > MS 11.35 ± 5.59; t = 2.077, *p* = 0.038); specifically, the MC students’ needs were greater than those of the MS students’. For wanted control, however, MS students showed greater needs (MS 5.49 ± 2.20 > MC 5.15 ± 2.29; t = −2.122, *p* = 0.034).

### Differences by academic level

Some differences were found between the school systems: MC students showed greater interpersonal needs than did the MS students (Table 3). Given that the MC students were generally younger, we wanted to confirm the result by analyzing the needs by academic level. The Levene’s test results showed homoscedasticity, allowing for using one-way ANOVA, which found differences in all categories except eI, wC, and C.

**Table 3:** FIRO-B need scores by academic level

		Academic level				F	*p*
		Premedical year 1	Premedical year 2	Medical year 1	Medical year 2		
Needs	I	8.56	7.77	7.21	7.05	7.205	<0.001
	C	8.87	8.76	8.41	8.38	1.581	0.192
	A	8.79	8.41	7.62	7.25	5.994	<0.001
Behaviors	e	12.75	12.32	11.03	11.16	6.499	<0.001
	w	13.47	12.62	12.21	11.51	4.625	0.003
Six dimensions of needs	eI	4.58	4.42	4.29	4.19	2.005	0.112
	eC	3.74	3.49	2.95	3.18	6.776	<0.001
	eA	4.43	4.41	3.79	3.79	4.266	0.005
	wI	3.98	3.35	2.92	2.86	8.257	<0.001
	wC	5.13	5.27	5.46	5.2	1.251	0.290
	wA	4.36	4.00	3.83	3.46	5.249	0.001
Overall		26.22	24.94	23.24	22.68	7.209	<0.001

Data are presented as means

*p*-value by one-way ANOVA

*I* inclusion, *C* control, *A* affection, *e* expressed, *w* wanted, *eI* expressed inclusion, *eC* expressed control, *eA* expressed affection, *wI* wanted inclusion, *wC* wanted control, *wA* wanted affection

## Discussion

There are many ways to promote and develop good relationships with patients, and one is to be aware of one’s interpersonal skills. Dyche observed that it is important for medical educators to focus at least somewhat on the verbal aspects of their relationship skills [9]. This study aimed to determine whether different approaches are needed for different students regarding their interpersonal needs. Because no studies to date have explored medical students’ interpersonal skills using the FIRO-B, it is difficult to compare this study’s results with those from previous studies. But FIRO-B can be a useful tool to assist and to enhance the students’ interpersonal and communication skills in medical education by acknowledging their behaviors in the context of dealing with people. This study’s results revealed moderate needs among the medical students for control, affection, and inclusion. It is interesting to see many students had high wA scores (56.3%) which means that they preferred not to show their affection but rather want others to show affection for them. Nearly as much students showing low wI scores (48.2%), meaning they would like to be asked to join groups or activities rather than initiating membership themselves, reveals that at leat half of the medical students prefer to be the recipients in the area of needs [10]. Expressed and wanted behaviors also showed moderate scores, indicating that the students had varying levels of control in interpersonal relations [10]. Of the six need dimensions on the FIRO-B, two showed differences by gender. Male students wanted more expressed control than did females, and females showed more wanted affection than did males. By specific need, inclusion, control, and affection, males showed higher control needs in their interpersonal relationships. This could be attributable to cultural differences that make males traditionally more dominant in the household in Korea [11], but it could also relate to how in junior high school, males and high academic achievers tend to lead their classes as representatives; however, this matter needs to be studied further. The male students’ greater desire for control in their interpersonal relationships reflects their increased desire to influence people, preference for situations with clear responsibility, desire for power, and desire to make decisions [10]. It may be necessary to acknowledge this fact with male medical students to ensure that they do not attempt to overly control their relationships with their patients and to remind them of the importance of patient-centered care. In contrast, females showed higher wanted affection, meaning they preferred intimate, warm, one-to-one relationships, to be supportive and open, to be considerate and caring, and to show sympathy. The female students also preferred that others express these behaviors toward them. This is an acceptable result because we generally consider females to be more affectionate than males.

A number of differences were found in the FIRO-B dimensions by school type and academic level, with the most significant differences being in expressed control and wanted inclusion; specifically, the needs in these areas were greater among the lower academic year students. Significant statistical differences were clearly seen between undergraduate freshmen and second-year graduate students. One previous study showed that participants who had high wanted control were less adjusted to work [12]. In Sharma’s study [13], expressed control had the highest correlation with one of five factors, conscientiousness, which relates to self-discipline and influences how we control our impulses. The level of conscientiousness increased among young adults and decreased with age [14]. These results are we would expect undergraduate freshmen to be very motivated to adjust to school, get involved in clubs and activities, and gain a sense of inclusion, whereas entering graduate students already have this experience and would thus be more comfortable when they enter their programs. However, further research is needed because these results do not exactly match the subject and the instrument of our study. It would be interesting to determine whether there exist any adjustment and self-confidence differences between undergraduate and graduate students, particularly looking at the correlations with the FIRO-B. Furthermore, tracking medical students’ satisfaction with their interpersonal relationships over the courses of their education would be valuable for mentoring and coaching them. Indeed, we were presented to the medical students by individual report. Especially Konyang University College of Medicine (KYUCOM) had a team projects during communication skills course or one as FIRO-B workshops. The students in KYUCOM enjoyed the FIRO-B sessions and workshop giving feedbacks such as they had valuable time to acknowledge their type of behaviors in communication and also the behaviors of others. Therefore we recommend to provide a 2 or 3 h education as a form of active class or as a workshop dealing with FIRO-B in other medical schools.

But in order to make the full use of FIRO-B instruments there needs to be some preparations. One of them is the training of some devoted faculty staff who can be trained to understand the instrument and then by faculty workshops within the institution, the advantages of FIRO-B can be widely spread. We also need give some brief orientation to the medical students the purpose and the benefit of FIRO-B. And when FIRO-B is used in actual scene, forming the students in small groups with similar types may help to share and understand the common characteristics. Thus small group tables with movable seating environment may help.

This study has limitations in generalizing its results because the subjects were limited to four medical schools in Korea. Therefore, more diverse study subjects and regions, including overseas, could give more interesting results. The study used the FIRO-B, and despite the originality of a study using that tool, future studies could expand to use other interpersonal relationship instruments, including comparing the results with those from the FIRO-B as in Dancer and Woods [15], Mahoney [16], and Sharma [13]. This has been attempted, but it is necessary to determine whether the FIRO-B could replace other instruments that are already widely used in medical schools.

## Conclusions

The FIRO-B is a useful tool for gaining insight into students’ interpersonal relationship orientations, which will help them to adjust to medical school life. FIRO-B data can be useful for mentoring and coaching students. Students’ academic levels, school systems, and gender can be taken into account when determining the instructional methods that effect greater control and responsibility, and also in developing more sophisticated education programs that include skills such as communication skills. And not only school life, we expect to improve performance to help other people understand when they meet patients as a physician or they are working as a team from the same organization. Merely helping medial students acknowledge their interpersonal behaviors will be helpful for building effective patient-physician relationships.

## Abbreviations

A: Affection
C: Control
e: Expressed
eA: expressed Affection
eC: expressed Control
eI: expressed Inclusion
FIRO-B: Fundamental interpersonal relations orientation – behaviour scale
I: Inclusion
MC: Undergraduate medical college
MS: Graduate medical school
W: Wanted
wA: wanted Affection
wC: wanted Control
wI: wanted Inclusion
